# p32 promotes melanoma progression and metastasis by targeting EMT markers, Akt/PKB pathway, and tumor microenvironment

**DOI:** 10.1038/s41419-021-04311-5

**Published:** 2021-10-28

**Authors:** Sunita Sinha, Satyendra Kumar Singh, Nitish Jangde, Rashmi Ray, Vivek Rai

**Affiliations:** 1 Laboratory of Vascular Immunology, Institute of Life Sciences, Bhubaneswar, 751023 India; 2 Manipal Academy of Higher Education, Manipal, Karnataka 576104 India

**Keywords:** Lung cancer, Cell migration

## Abstract

Melanoma originates from melanin-producing cells called melanocytes. Melanoma poses a great risk because of its rapid ability to spread and invade new organs. Cellular metastasis involves alteration in the gene expression profile and their transformation from epithelial to mesenchymal state. Despite of several advances, metastatic melanoma being a key cause of therapy failure and mortality remains poorly understood. p32 has been found to be involved in various physiological and pathophysiological conditions. However, the role of p32 in melanoma progression and metastasis remains underexplored. Here, we identify the role of p32 in the malignancy of both murine and human melanoma. p32 knockdown leads to reduced cell proliferation, migration, and invasion in murine and human melanoma cells. Furthermore, p32 promotes in vitro tumorigenesis, inducing oncogenes and EMT markers. Mechanistically, we show p32 regulates tumorigenic and metastatic properties through the Akt/PKB signaling pathway in both murine and human melanoma. Furthermore, p32 silencing attenuates melanoma tumor progression and lung metastasis in vivo, modulating the tumor microenvironment by inhibiting the angiogenesis, infiltration of macrophages, and leukocytes in mice. Taken together, our findings identify that p32 drives melanoma progression, metastasis, and regulates the tumor microenvironment. p32 can be a target of a novel therapeutic approach in the regulation of melanoma progression and metastasis.

## Introduction

Melanoma is identified as one of the most invasive form of skin cancer derived from mutated melanin-producing cells or melanocytes, resides predominantly in the skin. The early stages of melanoma are not lethal and can be treated by surgical removal. However, metastatic melanoma shows a poor prognosis and becomes challenging at advanced stages [1, 2]. Novel strategies like BRAF-targeted and immune therapies have played a critical role in improving the disease outcomes in recent years, but malignant melanoma still remains a perilous risk in humans [3]. Metastasis is a complex process that modulates cancer cell’s ability to infiltrate into the vasculature including lymphatic vessels, migrate, and colonize at distant tissues/organs [4, 5]. Cancer cells undergo metastatic transformation that involves various cellular and molecular changes including epithelial to mesenchymal transition (EMT) of cancer cells is one of important event that regulates cellular metastasis. Therefore, identification of biomarkers and the underlying mechanisms that regulate EMT will provide better insights of melanoma progression and metastasis [6].

p32, also known as p33/C1QBP, is a ubiquitous anionic cellular protein of 33 kDa [7] implicated in various pathophysiological conditions including inflammation and cancer [8–12]. p32 protein is a doughnut-shaped trimer located primarily in mitochondria, extracellular matrix, and intracellular compartments such as nucleus, Golgi apparatus, cytoplasm, and cell membranes and is involved in an array of ligand-mediated cellular responses [13]. p32 expression is found to be associated with various cellular processes, including autophagy and cancer. An elevated level of p32 protein has been found in the colon, pancreas, esophagus, and lung adenocarcinomas [14–16] and has also been reported to promote breast and prostate cancer [17]. Exogenous p32 promotes migration and invasion of melanoma cells [18]. Interaction of p32 with SAMMSON oncogene (long non-coding RNA) promotes pro-oncogenic function of melanoma cells [19]. p32 is associated with downregulation of migration and proliferation in cervical squamous cell carcinoma via the p38/MAPK signaling pathway [20]. Furthermore, a low level of p32 in renal carcinoma tissues indicates the pleiotropic role of p32 in tumor development [21]. However, the role of p32 in melanoma progression and metastasis remains poorly understood.

In this study, we show the role of p32 in melanoma cell tumorigenesis both in mice and humans. Further, p32 promotes tumorigenesis in mice and human melanoma cells by regulating the oncogenes expression, EMT markers, and Akt/protein kinase B (PKB) pathway activation. Furthermore, our studies in in vivo mice models show that p32 promotes tumor progression and metastasis by controlling the tumor microenvironment.

## Results

### p32 silencing attenuates invasion, colony formation capacity, migration, cell proliferation, and increase cell death in mice and human melanoma cells in vitro

**Fig. 1:**
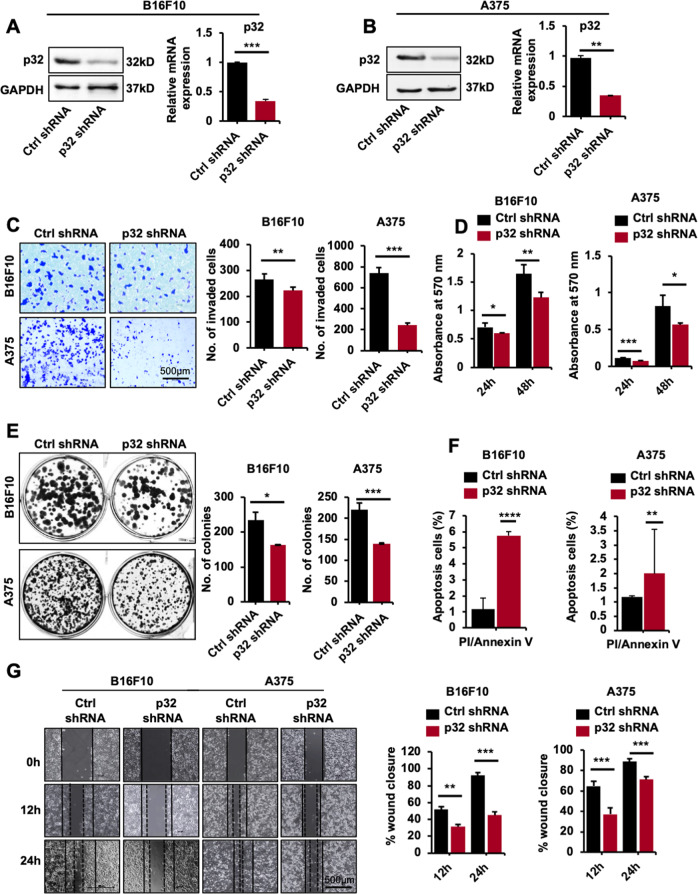
p32 silencing suppresses mouse B16F10 and human A375 melanoma cell tumorigenesis in vitro. **A** Immunoblot and relative mRNA expression showing the level of p32 in ctrl shRNA and p32 shRNA-treated B16F10 cells. **B** Immunoblot and relative mRNA expression showing the level of p32 in ctrl shRNA and p32 shRNA-treated A375 cells. **C** Invasion assay showing the number of invading cells through transwell chamber 12 h after stimulation with 5% FBS; **D** cell proliferation quantified by MTT assay after 24 and 48 h; **E** colony formation assay showing colony numbers per well along with graphical representation. **F** Bar graph quantifying the percentage of annexin V and PI staining in B16F10 and A375 cells and **G** wound-healing showing 0, 12, and 24 h post wounding and its graphical representation showing percentage wound closure in ctrl shRNA and p32 shRNA-treated B16F10 and A375 cells. All data are represented as mean ± SD. Student’s *t*-test was used for all statistical analyses, *n* = 3 (****P < 0.0001,***P < 0.001, **P < 0.01, *P < 0.05).

Cell proliferation and the capability of a single tumor cell to grow into a colony are important characteristics of the cancer cells to evaluate its tumorigenesis potential in vitro [22]. In order to identify the role of p32 in melanoma, we evaluated the expression levels of p32 in three different human melanoma SK-MEL-2, SK-MEL-28, and A375 cell lines through immunofluorescence (Supplementary Fig. 1) and further validated with western blot analysis (Supplementary Fig. 2A) and measuring transcript levels (Supplementary Fig. 2B) of p32 in these cells. Our results showed the highest and profound expression of p32 in A375 cells. Next, to identify the oncogenic properties, we checked the expressions of oncogenes and EMT markers and our results show the higher expression of oncogenes and EMT markers in A375 cells compared to SK-MEL-2 and SK-MEL-28 (Supplementary Figs. 3 and 4). Based on the highest expression of p32, oncogenes, and EMT markers, human A375 cells were selected for further p32 studies in melanoma and also the A375 cells are well reported for the most aggressive melanoma [23] and murine B16F10 cell line, well known effective murine tool for metastasis research. To find out the possible role of p32 in melanoma tumorigenesis in vitro, we silenced p32 in murine melanoma B16F10 cells and human melanoma A375 cells using specific p32 shRNA and further our immunoblot and transcript level analysis results showed profound inhibition of p32 in both the murine (Fig. 1A) and human melanoma cells (Fig. 1B). Cell proliferation, invasion, and the capability of a single cell to grow into a colony are important characteristics of the cancer cell to evaluate its tumorigenesis potential in vitro [22]. To study the involvement of p32 in invasiveness and proliferative capacity of these cells, we performed transwell invasion and cell proliferation assay and results showed p32 knockdown decreased invasion and proliferation of B16F10 and A375 cells (Fig. 1C, D). Next, clonogenic assay was performed to study the impact of p32 on colony formation, and the result showed small and a fewer numbers of colonies in p32 silenced B16F10 and A375 cells compared to control cells, respectively (Fig. 1E). We next determined whether p32 silencing also leads to increase cell death in melanoma cells. Our results showed that the percentage of annexin V and PI cells were higher in p32 silenced B16F10 and A375 cells compared to control (Fig. 1F and Supplementary Fig. 5A). In addition, p32 knockdown in melanoma cells showed an increased cleaved PARP and caspase 9 level which are hallmark of apoptosis (Supplementary Fig. 5B), Suggesting the inhibition of p32 expression in melanoma cells reduces the tumorigenicity by increasing the apoptosis. Further, we investigated the role of p32 in migration/wound healing and found that p32 silencing inhibited the migration of B16F10 and A375 cells (Fig. 1G). Together, the in vitro findings suggest the active role of p32 in melanoma tumorigenesis.

### p32 regulates expression of oncogenes and EMT markers in murine and human melanoma cells

**Fig. 2:**
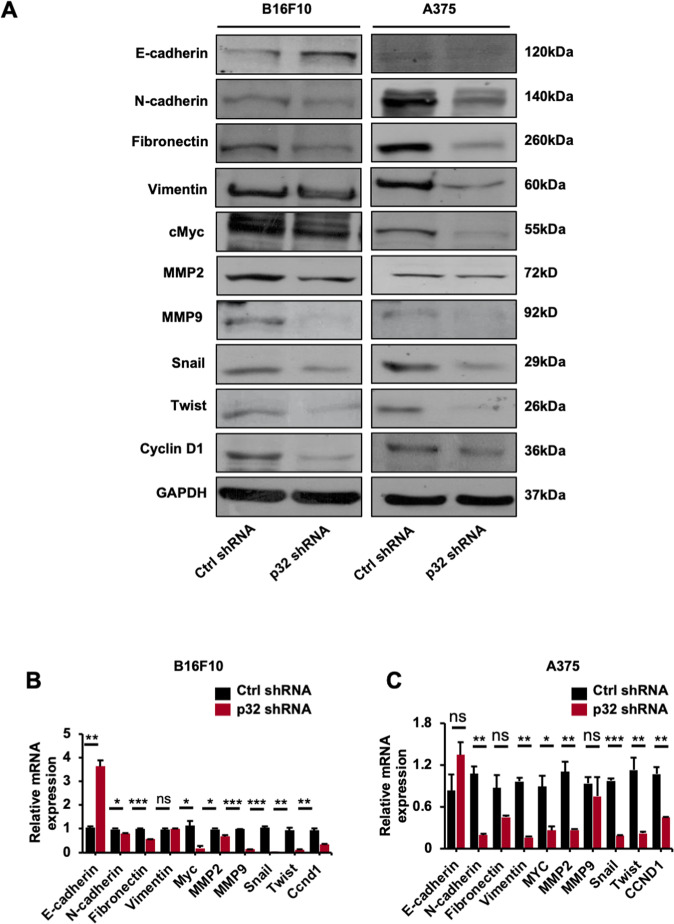
p32 silencing decreases the expression level of oncogenes and EMT markers both in mouse and human melanoma cells in vitro. **A** Immunoblots showing the expression level of oncogenes and EMT markers and **B, C** relative mRNA level of various oncogenes and EMT markers in ctrl shRNA and p32 shRNA-treated B16F10 and A375 cells, respectively. All the data are represented as mean ± SD. Student’s *t*-test was used for all statistical analyses, *n* = 3 (***P ≤ 0.001, **P ≤ 0.01, *P ≤ 0.05).

Studies have shown that EMT markers and oncogenes expression regulate in vitro cancer cell tumorigenic properties [22, 24] and our results showed that p32 silencing inhibited the cell proliferation, migration, colony formation capacity, and invasion in B16F10 and A375 cells. Next, we investigated if p32 silencing alters oncogenes expression, EMT mediators and downstream signaling pathways implicated in B16F10 and A375 cells in vitro tumorigenesis. Our immunoblot and qPCR results showed that the p32 downregulation increased expression of epithelial marker (E-cadherin) in B16F10 cells. Furthermore, p32 silencing decreased the expression of mesenchymal markers including matrix metalloproteins (MMP2, MMP9, N-cadherin, fibronectin, and vimentin) in both B16F10 and A375 cells and Snail and Twist1 in both cell types, p32 downregulation also decreased oncogenes expression (cMyc and cyclin D1) in both B16F10 and A375 cells (Fig. 2A–C) and quantification of immunoblots analysis (Supplementary Fig. 6A, B).

**Fig. 3:**
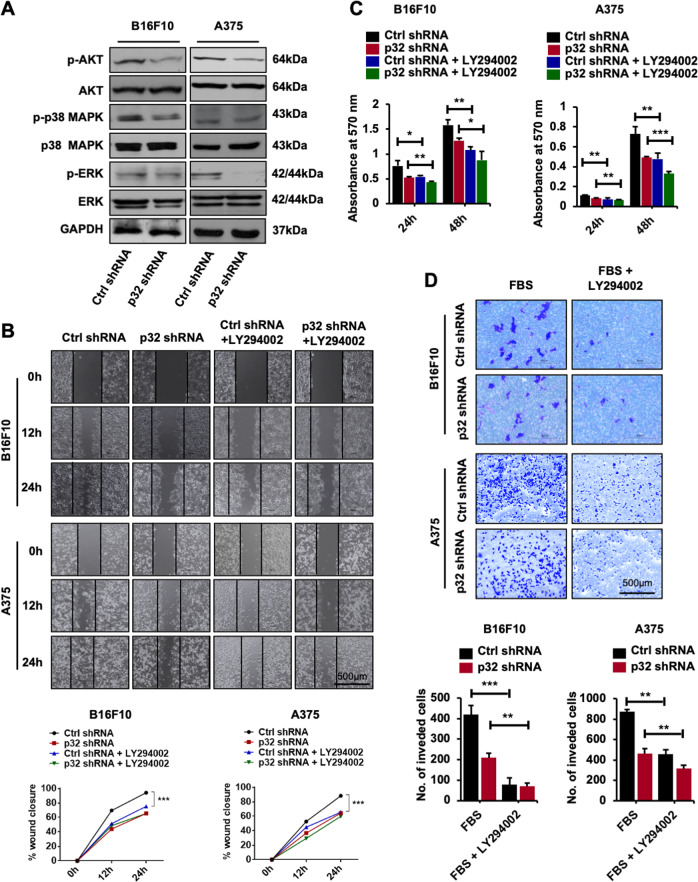
p32 silencing induces melanoma cell tumorigenesis via Akt/PKB pathway in melanoma cells. **A** Immunoblot showing the phosphorylation levels of p-Akt, p-p38, and p-ERK in B16F10 and A375 melanoma cells after p32 silencing. **B** Wound-healing/scratch assay showing cell migration; **C** cell proliferation at 24 and 48 h and **D** invasion assay showing the number of invading cells through transwell chamber 12 h after stimulation with 5% FBS in ctrl shRNA and p32 shRNA-treated B16F10 and A375 cells with and without Akt/PKB inhibitor LY294002 treatment. All the data are represented as mean ± SD. Student’s *t*-test was used for all statistical analyses, *n* = 3 (***P < 0.001, **P < 0.01, *P < 0.05).

p32 is known to modulate the cellular properties and disease pathogenesis through various downstream signaling pathways (ERK1/2, p38, and Akt) [20, 21, 25, 26]. To identify the signaling pathways that mediate the p32-dependent in vitro tumorigenesis in melanoma cells, the phosphorylation levels of kinases were evaluated by immunoblot analysis in control and p32 shRNA transfected B16F10 and A375 cells. p32 knocking down decreased the phosphorylation levels of Akt both in B16F10 and A375 cells and ERK in A375 cells as shown in immunoblot (Fig. 3A) and their densitometric quantification (Supplementary Fig. 7). These results suggest an involvement of oncogenes, EMT markers, and downstream signaling pathways in p32-mediated tumorigenesis in melanoma cells.

### p32 promotes murine and human melanoma cells tumorigenesis via the Akt/PKB pathway

p32 knockdown decreased the activation of p38 in murine cells, ERK in human cells, and Akt/PKB in both melanoma cell types. So, we evaluated the involvement of p32-Akt/PKB axis in melanoma cells tumorigenesis. Next, to identify if the p32 is involved in in vitro tumorigenesis via the Akt/PKB signaling pathway in B16F10 and A375 cells, we pre-treated control and p32 silenced murine and human melanoma cells with phosphoinositide 3-kinase (PI3K) inhibitor LY294002 and confirmed that LY294002 treatment significantly inhibited Akt/PKB activation in both B16F10 and A375 cells (Supplementary Fig 8A). Further, we assessed cell migration, proliferation, and invasion after Akt/PKB inhibitor LY294002 treatment in murine and human melanoma cells. Our results showed a decrease in cell migration at 12- and 24-h time points (Fig. 3B), proliferation (Fig. 3C), and invasion (Fig. 3D) in cells treated with inhibitor as compared to control cells there was little decrease in proliferation and invasion but no significant change in migration in p32 shRNA cells after Akt inhibitor LY294002 treatment suggesting p32-dependent Akt activation mainly leads these functions in melanoma. Next, we explored the effect of Akt inhibitor on EMT markers and the oncogenes in both murine and human melanoma cells. Our qPCR results showed decreased expression of EMT markers including matrix metalloproteins (E-cadherin, N-cadherin, fibronectin, vimentin, Snail, Twist1, MMP2, and MMP9), and oncogenes (cMyc and cyclin D1) in both the murine and human melanoma control cells treated with inhibitor but there was no significant change in the p32 shRNA with LY294002 treatment as compared to p32 shRNA silenced mouse and human melanoma cells (Supplementary Fig. 8B, C). These results confirm that p32 promotes in vitro melanoma tumorigenesis via the Akt/PKB pathway.

### p32 promotes murine melanoma progression and modulates tumor microenvironment in vivo

**Fig. 4:**
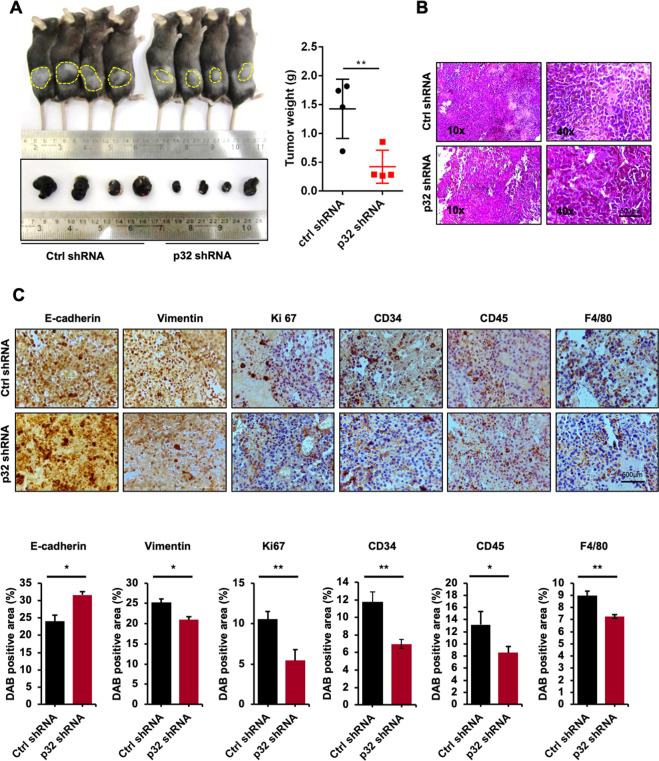
p32 knockdown in mouse melanoma cells impairs their tumor-forming capacity and modulates tumor microenvironment in vivo. **A** Representative images showing subcutaneous tumors size and weight and **B** representative H&E images of tumor section in C57BL/6 mice injected with control and transfected B16F10 cells subcutaneously. **C** Immunohistochemical analysis of E-cadherin, vimentin, proliferation (Ki-67), angiogenesis (CD34), leukocyte (CD45), and macrophage (F4/80) infiltration in subcutaneous tumors and bottom panel bar graphs represent their quantification. All the data are represented as mean ± SD. Student’s *t*-test was used for all statistical analyses, *n* = 5 (**P < 0.01, *P < 0.05).

To further investigate the role of p32 in tumor progression in vivo, we injected control shRNA and p32 shRNA-treated B16F10 cells subcutaneously in C57BL/6 mice and after 20 days mice were euthanized. Tumor size and weight were measured. Mice showed effectively reduced tumor formation (size and weight) injected with p32 shRNA-transfected cells in comparison to control shRNA-transfected cells as shown (Fig. 4A) and to identify the pattern, shape, and structure of cell in these tumors, H&E staining of these tissue sections was performed (Fig. 4B). Next, our immunohistochemical analysis results showed decreased level of pAKT in the tumors formed by p32 shRNA-transfected cells as compared to the tumors formed by control shRNA-transfected cells (Supplementary Fig. 9A). Furthermore, our qPCR results showed that the tumors formed by p32 shRNA-transfected B16F10 cells show increased expression of epithelial marker (E-cadherin) and decreased expression of mesenchymal markers (N-cadherin, fibronectin, and vimentin), Snail, and Twist as compared to the tumors formed by control shRNA-transfected B16F10 cells (Supplementary Fig. 9B). The tumor microenvironment plays an important role in cancer cell survival. The two-way communication between tumor cells and non-resident cells regulates tumorigenesis and metastasis [27]. Further to investigate immune cell regulation, immunohistochemical analyses were performed in tumor tissue sections to study the effect of p32 knocking on the expression of E-cadherin, vimentin, proliferation marker Ki-67, tumor angiogenesis marker CD34, leukocytes maker (inflammatory cell recruitment, CD45), and macrophage marker F4/80. p32 silenced B16F10 cells subcutaneous tumors showed decreased proliferation (Ki-67), angiogenesis (CD34), leukocyte (CD45), macrophage (F4/80) markers, and vimentin expression than control tumors (Fig. 4C) confirming its involvement with p32-mediated melanoma tumor progression.

### p32 regulates lung metastasis controlling tumor microenvironment of melanoma

**Fig. 5:**
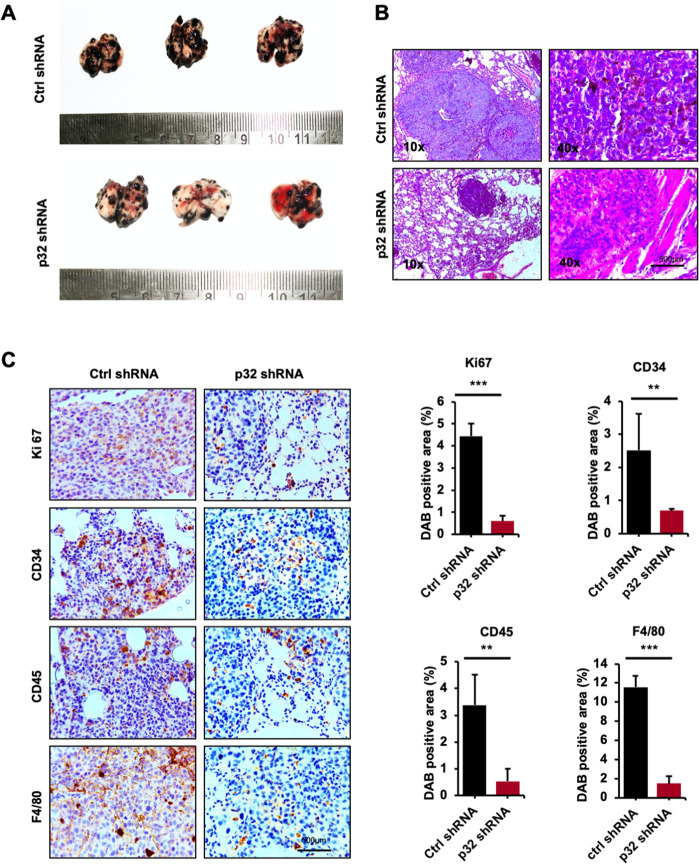
p32 knockdown impairs mice melanoma cells lung metastasis and modulate associated tumor microenvironment in vivo. **A** Representative images showing tumors in lung after intravenous injection of control and transfected B16F10 cells**. B** Representative H&E images of lung tissue sections showing tumors in C57BL/6 mice injected with control and transfected B16F10 cells intravenously. **C** Immunohistochemical analysis of proliferation (Ki-67), angiogenesis (CD34), leukocyte (CD45), and macrophage (F4/80) infiltration in intravenous tumors from C57BL/6 mice after injecting control and shRNA-transfected B16F10 cells and right panel bar graphs represent their quantification. All the data are represented as mean ± SD. Student’s *t*-test was used for all statistical analyses, *n* = 5 (***P < 0.001, **P < 0.01).

Next, to study the role of p32 in melanoma cells lung metastasis, we injected control and p32 shRNA-transfected B16F10 cells intravenously through the tail vein in C57BL/6 mice, and our results showed that the p32 shRNA-transfected B16F10 cells developed a very less numbers as well as small tumor foci in lungs (Fig. 5A) and their H&E staining of lung tissue sections showing the tumors formation in these tissues (Fig. 5B).

In agreement to our tumor progression observation, in metastasis study, lung tumors sections showed the lower expression of cell proliferation marker Ki-67 and angiogenesis markers CD34 in p32-silenced B16F10 cells (Fig. 5C). Furthermore, leukocytes (CD45) and macrophage (F4/80) infiltration were also lower in p32-silenced B16F10 cell tumors as represented with their quantification (Fig. 5C).

### Association of p32 expression with melanoma metastasis and patient survival

**Fig. 6:**
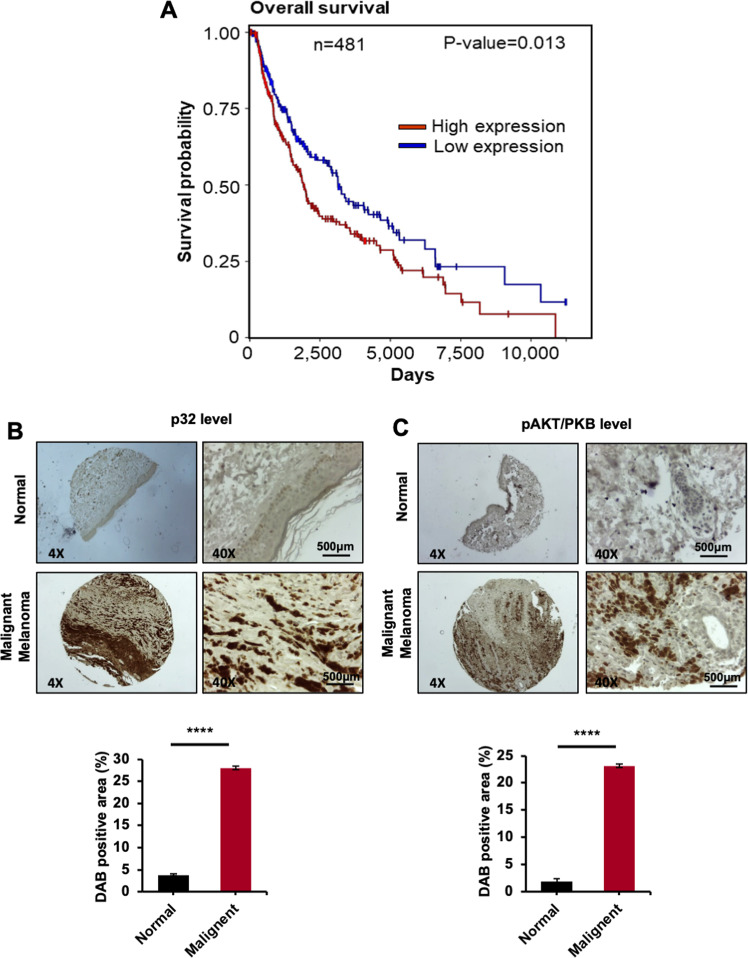
Assessment of the overall survival according to p32/c1qbp expression in patients with melanoma. **A** Survival plot data (Kaplan–Meier plot; UCSC Xena browse tool) downloaded from TCGA Melanoma datasets illustrates better survival among melanoma patients (*n* = 481) with lower p32/c1qbp expression levels. **B** Immunohistochemical analysis of p32 in human melanoma tissue microarray containing normal and malignant melanoma tissue sections. **C** Immunohistochemical analysis of pAKT in human melanoma TMA containing normal and malignant melanoma. All data are represented as mean ± SD. Student’s *t*-test was used for all statistical analyses, *n* = 3 (****P < 0.0001).

**Fig. 7:**
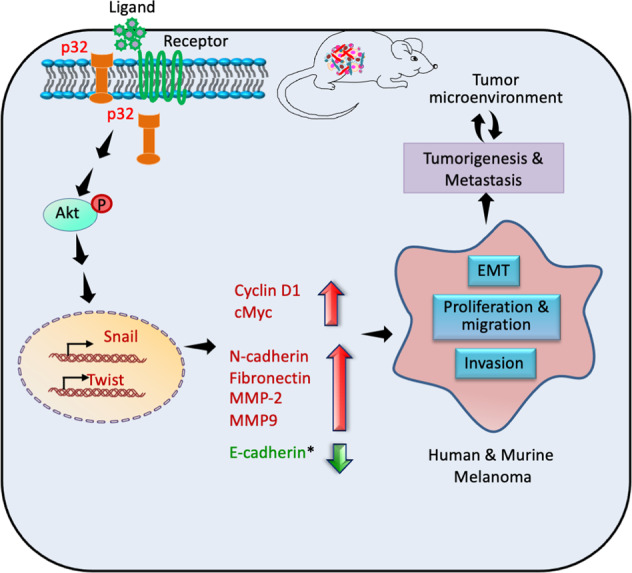
Schematic demonstrating p32 regulates melanoma via AKT/PKB signaling. **A** Schematic representation showing that p32 regulates melanoma tumorigenesis and metastasis via modulating Akt/PKB signaling, EMT markers, oncogenes expression, and tumor microenvironment (* in mouse melanoma).

To investigate the association between p32 expression and survival in patients with melanoma, Kaplan–Meier survival analysis was done of 481 samples using data from the TCGA databases from the UCSC Xena browser and Kaplan–Meier plotter tools. Our analysis showed that the overall survival was associated with lower p32/c1qbp expression in patients with melanoma (Fig. 6A). To further validate the role of p32 in promotion of murine and human melanoma cells tumorigenesis, we examined the expression level of p32 in human normal and malignant melanoma skin tissues and we found p32 is highly expressed in malignant melanoma skin tissue as compared to the normal skin tissue (Fig. 6B). Furthermore, we also determined the Akt/PKB activation in human normal skin tissue and malignant melanoma skin tissue and found higher activation of Akt/PKB in malignant melanoma skin tissue compared to normal skin tissue (Fig. 6C). These human melanoma results corelate our findings that p32 regulates melanoma tumorigenesis and metastasis via modulating Akt/PKB signaling in mice melanoma, EMT markers, oncogenes expression, and tumor microenvironment detailed as a schematic diagram illustrated (Fig. 7).

## Discussion

p32 has been identified as a mediator of various pathological conditions, including cancers. Though it is found to be involved in regulating tumor progression, its role in melanoma tumorigenesis and metastasis is not well understood. Here, our results establish that p32 regulates in vitro malignancy in both murine and human melanoma. In our study, we used B16F10 mouse melanoma cells, a well known effective murine tool for metastasis research and A375 human melanoma cells on the basis of higher levels of p32 as comparison to the other two cell lines SK-MEL-2 and SK-MEL-28. In addition, A375 cells are well reported for the most aggressive melanoma cells were selected. p32 silencing inhibits cellular proliferation, migration, and invasion in murine (B16F10) and human (A375) melanoma cells. Furthermore, we have shown that p32 silencing inhibits B16F10 cell tumor development and lung metastasis. Overall, data from this study confirm that p32 promotes melanoma cells tumorigenesis majorly by modulating the Akt/PKB signaling pathway, EMT transition, and manipulating the tumor microenvironment.

p32 (33 kDa) is a multi-functional protein ubiquitously expressed in cells and is upregulated in various cancers, including mammary cancer tissues, and promotes metastasis and progression to advanced stages [14–16]. It also regulates lamellipodia formation and metastasis in human lung carcinoma A549 cells [26]. Studies have shown that knocking down p32 impairs cellular growth in cancer cells MDA-MB-435 [28–32]. Furthermore, C1qbp silencing in MDA-MB-231 breast cancer cells reduces proliferation, migration, and induces doxorubicin-induced apoptosis [24]. Although studies on other cancers also suggest an involvement of p32 in tumorigenesis and metastasis, recently it has been shown that targeting gC1qR at the C1q-binding site can significantly reduce mesothelioma tumor burden by increasing tumor cell apoptosis and decreasing tumor angiogenesis [33] and ablation of p32 induces apoptosis in colorectal cancer cells [34] but its role in melanoma remains mostly unknown. Previous studies have shown the involvement of exogenous p32 protein in migration and invasion of B16F10 melanoma cells [18] and interaction of p32 with SAMMSON oncogene promoting pro-oncogenic function [19]. Our study has shown that knocking down p32 suppresses the various in vitro tumorigenic properties of the murine and human melanoma cells, including cell proliferation, migration/wound-healing capacity, clonogenic potential, and invasiveness. Taken together, our data establish that p32 promotes tumorigenic properties of murine and human melanoma cells.

Snail and Twist are the hallmarks oncogenic transcription factors that downregulate the expression of epithelial marker E-cadherin and upregulate EMT markers fibronectin, c-Myc, cyclin D1, MMP2, and MMP9 in various cancers [35–38]. In the present study, we provide evidence that p32 promotes various carcinogenic properties of murine melanoma cell, and the expression of oncogenic transcription factors Snail, Slug, and Twist together with various key mesenchymal markers (N-cadherin, fibronectin, MMP2, and MMP9) and oncogenes (c-Myc and cyclin D1) and further validation on human melanoma cells (A375) signifies the involvement of p32 in melanoma progression, and metastasis. Our present data indicate that p32 promotes melanoma cells’ tumorigenic properties via upregulation of oncogenes and mesenchymal markers, and loss of epithelial markers.

p32 mediates tumor development by regulating the migratory and invasive properties of cancer cells through various downstream signaling pathways, including p38 MAPK and GSK3/β-Catenin/L1CAM [20, 21]. Next, we evaluated the phosphorylation levels of signaling phospo-proteins and found less phosphorylation of ERK/MAPK proteins on p32 silencing in human melanoma cells but significant reduction in phosphorylation level in Akt in both mouse and human melanoma cells. Earlier studies on lung and breast cancer cells have shown reduced activation of Akt and ERK in both cell surface neutralization and shRNA-based silencing of p32 in A549 and MDA-MB-231 cells [25, 26]. We further validated the Akt signaling pathway’s involvement in p32-mediated tumorigenesis using AKT inhibitor LY294002 in melanoma cells and found that p32-mediated in vitro tumorigenic properties of both murine B16F10 and human A375 melanoma cells are Akt-dependent. Our present data indicate that p32 drives tumorigenic properties of melanoma cells via Akt-dependent cell proliferation, invasion and migration, confirming Akt/PKB as a major signaling pathway involved in p32-mediated melanoma tumor progression and metastasis.

It has been reported that p32 promotes tumor development in glioma cells, breast cancer, and lung cancer cells [26, 29, 39]. Our study on murine melanoma cell has identified that p32 promotes the development of large size and weight of tumor in the subcutaneous mice model. In addition, in vivo metastasis experiment showed that p32 promotes melanoma cell metastasis showing larger tumor foci in lungs. These in vivo findings give a new dimension to p32 studies in cancers, specifically in melanoma progression and metastasis, strongly advocating p32 as a tumor promoter and its role in melanoma tumor development and metastasis.

The tumor microenvironment consists of resident tumor cells and non-resident immune cells together with connective tissues play an important role in tumor development and metastasis [40]. Understanding the role of p32 in the regulation of mutual communication between tumor cells and their microenvironment can be an attractive therapeutic approach to target different mechanisms that promote tumor progression. Our data show that p32 knockdown strongly inhibits angiogenesis, proliferation markers, and further infiltration of leukocytes and macrophages to subcutaneous and lung metastasized melanoma tumors. Reports have established the involvement of macrophages and angiogenesis markers in tumor progression, and metastasis in various cancers [41–43]; however, future studies on p32 and tumor microenvironment, including immune cell types and their orientation, will be critical to find out the precise mechanisms involved in melanoma tumorigenesis. Furthermore, understanding how the presence of p32 in tumor cells regulates the communication between tumor cells and their microenvironment to drive in vivo tumorigenesis will be important. Our study shows that p32 is highly upregulated in melanoma patients as compared to the normal skin tissues. We found significant decrease in the level of p-Akt in both murine and human melanoma cells on p32 silencing. Furthermore, Akt/PKB inhibitor LY294002 treatment show decrease in the expression of twist which upholds epithelial to mesenchymal transition [44] and snail which stimulates cell migration [45]. Collectively our data supports that the p32 plays a critical role in melanoma progression in both murine and human cells by repression of various EMT markers and oncogenes via Akt/PKB pathway which is known to alter EMT markers and oncogenes in other cancers [44, 45]. Taken together, our data indicate p32 as a mediator of melanoma tumorigenesis and its metastasis to the lung by regulating the melanoma cells themselves and the associated tumor microenvironment.

In conclusion, our study identifies p32 as a tumor promoter in melanoma tumorigenesis and metastasis by promoting various tumor cell properties via the Akt/PKB pathway, altering EMT markers in both mice and human melanoma. Further, p32 promotes melanoma tumor progression and metastasis in mice. Furthermore, p32 regulates higher macrophages and leukocytes infiltration in the tumor milieu, providing evidence that it manipulates the tumor microenvironment to facilitate melanoma progression and metastasis. However, further studies are required to elaborate the role of p32 in tumor microenvironment regulation. Taken together, our study indicates that p32 could be a unique future therapeutic target for controlling the progression and metastasis of melanoma.

## Materials and methods

### Animals

All animal were bred and maintained in animal house facility of Institute of Life Sciences. Animal protocols were approved by the Institute of Life Sciences Animal Ethics Committee. All animals used in the experiments were of C57BL/6 background (male) and 6–8 weeks of age.

### Cell lines and cell culture

B16F10 (mouse) and A375, SK-MEL-2, and SK-MEL-28 (human) melanoma cells were cultured in Dulbecco’s modified Eagle’s medium (DMEM) supplemented with 10% FBS, streptomycin (100 μg/ml), and penicillin (100 unit/ml). Cells were maintained in an incubator with a humidified atmosphere containing 5% CO_2_ at 37 °C.

### p32 lentiviral infection

High-titer p32 lentiviral particles from (TRCN0000066593 for mouse, TRCN0000300658 for humans; Sigma-Aldrich) and control shRNA lentiviral particles [46] (Santa Cruz Biotechnology) were used to knock down p32 in B16F10 and A375 cells. Cells were cultured in 96-well plates and infected with control shRNA, p32 shRNA in Opti-MEM. shRNA-infected cells were left for 8 h at 37 °C and 5% CO_2_. The medium was replaced with the fresh medium for 24 h. After 24 h of resting control shRNA and p32 shRNA-transfected cells colonies were selected from cells grown in medium with 1 µg/ml puromycin. The puromycin-selected colonies were evaluated for p32 silencing using western blotting for p32 expression.

### Inhibitor treatment

Cells were treated with 5 µM of Akt inhibitor (LY294002) for 1 h for cell migration, proliferation, and cell invasion assays.

### Cell invasion assay

The cell invasion assay was performed using six-well transwell units (SPL inserts) with an 8-µm pore size polycarbonate filter. The transwells were coated with 1 mg/ml type I collagen and incubated at 4 °C overnight. After washing with PBS, the wells were seeded with cells at a concentration of 2 × 10^5^ cells/ml in incomplete DMEM medium, and the bottom chambers were filled with 500 µl of cell medium containing 5% FBS. Cells were allowed to migrate overnight for 16 h at 37 °C and 5% CO_2_. After incubation, cells on the top surface of the membrane (non-migrated cells) were scraped with a cotton swab, whereas cells on the bottom side of the membrane (migrated cells) were stained with 0.5% crystal violet dye in 10% formalin for 30 min. The cells were then destained in PBS, and the membrane was left to air dry at room temperature. Migrated cells were counted using a Carl Zeiss inverted microscope (Carl Zeiss, Germany). Six independent areas per filter were counted, and the mean number of migrated cells was calculated.

### Cell proliferation assay

Cell viability assay was performed to check the proliferation rate of B16F10 and A375 cells and cells were seeded in 96-well cell culture plastic plates at a density of 5 × 10^3^ cells/well. After incubation for 48 h, 100 μl of 3-(4,5-fimethylthiazol-2-Yl)-2,5-diphenyltetrazolium bromide 5 mg/ml for 4 h and formazan crystals were dissolved by adding dissolving solution (DMSO: isopropanol; 1:1) for 30 min. The absorbance of each well was acquired at 570 nm with the help of an ELISA plate reader.

### Clonogenic assay

Clonogenic assay was performed to measure the growth ability of B16F10 and A375 single cell to grow into a colony in vitro. Briefly, after transfecting B16F10 and A375 cells with p32 shRNA or scrambled control cells were seeded in complete DMEM media at a density of 5 × 10^2^ cells in six-well plates. The plates were incubated for 2 weeks at 37 °C and then stained with 0.5% crystal violet. Colonies with greater than 50 cells were counted manually.

### Wound-healing assays

Cell migration ability of melanoma cells on 5% FBS treatment was detected by scratch assay. Cells were seeded in 12-well plates at the density of 3 × 10^5^ cells/well. After 24 h when cells reached 90–100% monolayer confluence, they were serum starved overnight. Post overnight serum starvation, a straight scratch was artificially created in the cell monolayers with 200 μl sterile pipette tip. Cells debris by the scratch was removed with phosphate-buffered saline (PBS), and cultures were then supplemented with DMEM medium alone and with 5% FBS for 24 h at 37 °C. Migration images were captured using an inverted microscope (Carl Zeiss, Germany). The scratch wound widths were measured under a microscope and the relative percentage of wound closure was determined by comparing to control cells.

### Quantitative PCR

Total RNA from ctrl shRNA and p32-shRNA-treated B16F10, A375, SK-MEL-2, and SK-MEL-28 cells and tissue were isolated using Trizol (Invitrogen) and further purified by pure link RNA Mini Kit (Ambion). Next, 2 μg of RNA was reverse transcribed using a High-Capacity cDNA Reverse Transcription Kit (Applied biosystems). Real-time PCR amplification was performed using SYBR green (Applied biosystems) and QuantStudio 6 flex Real-Time PCR (Applied Biosystem). A complete list of PCR primers is shown in Supplementary Table 1. All data were normalized to the housekeeping gene GAPDH.

### Immunoblot analysis

Total protein extract was obtained by homogenizing B16F10 and A375 cells in cell lysis buffer (Cell Signaling) added with 1 mM PMSF and 1 mM sodium fluoride [47] and incubated at 4 °C for 30 min, centrifuging for 1 min every 10 min. Cell lysates were centrifuged (12,000*g*, 15 min at 4 °C) to remove the cell debris, and the supernatant was collected and stored at −80 °C for further use. Protein concentration was determined using the Bradford protein assay method. Total protein extracts (40 μg) were then electrophoresed in 10% SDS-PAGE gels. Proteins were transferred to nitrocellulose membrane, and membranes were blocked in 1× Tris-buffered saline (TBS) and 0.1% Tween-20 (TBST) with 5% non-fat milk at room temperature for 1 h, followed by overnight incubation with diluted primary antibody (Supplementary Table 2) in blocking buffer at 4 °C with gentle shaking. After washing with TBST, the membrane was incubated at room temperature for 1 h with polyclonal anti-rabbit and anti-mouse IgG secondary antibodies conjugated to horseradish peroxidase (HRP) (Amersham, 1:5000 dilution). Membranes were visualized with enhanced chemiluminescence, followed by exposure to film.

### In vivo tumorigenesis

C57BL/6 mice (6–8-weeks-old) were maintained in specific pathogen-free conditions. All experimental protocols were approved by the Animal Ethics Committee of Institute of Life Sciences and all experiments were performed in accordance with the approved guidelines and regulations. For the subcutaneous tumor model, B16F10 cells were transfected with p32 shRNA or control shRNA and suspended with PBS at a concentration of 2 × 10^6^ cells/200 μl, and subcutaneously injected into C57BL/6 mice (*n* = 4 for each group). The mice were observed for 2 weeks.

For lung metastasis study, control and p32 shRNA-transfected B16F10 cells were suspended with PBS at a concentration of 1 × 10^6^ cells/200 μl and intravenously injected into C57BL/6 mice (*n* = 3 for each group). At the end of the experiment (after 3 weeks), mice were euthanized, and the lungs were collected. The B16F10 tumor specimens were fixed in 10% formalin, embedded in paraffin, and sectioned at 5 µm for histopathological studies.

### Immunofluorescence and confocal microscopy

For, immunofluorescent staining, cells were grown on cover glass were fixed at room temperature for 5 min in 100% methanol (chilled at −20 °C). After fixation, cells were washed with PBS and permeabilized with permeabilization buffer (PBS containing 0.1% Triton X-100). Nonspecific binding of antibody was blocked by incubation with 1 % bovine serum albumin (BSA) for 30 min at room temperature. Cells were incubated with the primary antibody in 1% BSA in PBST in a humidified chamber overnight at 4 °C, then washed and stained with DAPI and conjugated secondary antibodies. After mounting with Prolong Antifade gold reagent (life technologies), the cells were visualized under a Leica CTR6500 confocal microscope (Leica microsystems Wetzlar, Germany).

### Histopathology and immunohistochemistry

Immunohistochemical staining were carried out using tumor tissue samples and tissue microarrays purchased from Novus (NBP2-78131), In brief, formalin-fixed, paraffin-embedded sections, including 16 tumor tissues of melanoma, 6 tumor tissues of normal and nonmelanoma tumor tissues of the skin in duplicates. Tumor tissues samples were fixed in 10% formaldehyde solution before being embedded in paraffin wax. Paraffin-embedded tissue were sectioned into 5 µm slices and mounted on positively charged slides. Then the sections were deparaffinized in xylene and hydrated (100% EtOH, 95% EtOH, 70% EtOH, 2× H_2_O (2 min each) 3× distilled H_2_O (2 min each)). Then the sections were stained with Harris’ haematoxylin solution and washed with distilled water. Then sections were counterstained with eosin followed by dehydration and xylene and DPX mounting. For immunohistochemical assay, 3% hydrogen peroxide in methanol was used for endogenous peroxidases blocking. These sections were probed with E-cadherin (SC-7870), Vimentin (3932 S), Ki-67 (Ab15580), CD34 (sc-7324), CD45 (M0701, Dako), and F4/80 (sc-26643) primary antibody overnight at 4 °C. Tumor tissue sections slides were subsequently incubated with HRP-tagged secondary antibody. Staining was visualized with DAB and counterstained with haematoxylin. The images were taken by using a Leica microscope and image intensity was quantified using ImageJ software.

### Annexin V and propidium iodide staining

Apoptosis was assayed using the Annexin V–propidium iodide (PI) method with a commercially available kit (Cell Signaling Technology) according to the manufacturer’s instructions. Control shRNA and p32 shRNA-transfected B16F10 cells and control shRNA and p32 shRNA-transfected A375 cells were seeded in 10 cm^2^ cell culture plates at 70–80% confluency; cells were harvested washed twice with cold phosphate-buffered saline (PBS) and resuspended in Annexin binding buffer according to the manufacturer’s protocol. Cells were labeled by adding Annexin V-FITC and PI in each sample. Cell suspension was mixed and incubated for 10 min on ice in the dark; samples were analyzed on a flow cytometer (Accuri C6, BD Biosciences) for the detection of Annexin V and PI-positive subpopulations. All tests were performed in duplicates and experiments were repeated twice.

### Kaplan–Meier survival analysis

Association between p32/c1qbp expression and melanoma patient survival was assessed using the TCGA (The Cancer Genome Atlas) dataset. Survival data of melanoma patients were obtained using UCSC Xena Browser (http://xenabrowser.net). UCSC Xena Browser showed that TCGA dataset contained *n* = 481 cases of melanoma with c1qbp expression and survival rate information, and we generated a Kaplan–Meier survival analysis to analyze the relationship between p32/c1qbp gene expression and overall survival in melanoma patients.

### Statistical analysis

Data are presented as mean ± standard deviation (SD) and analyzed using Microsoft Excel and Graphpad Prism6.0 (GraphPad Software, La Jolla, CA). Statistical analyses were conducted between the controls and the treated experimental groups using Student’s *t*-test assuming two-tailed distributions, and differences were considered to be statistically significant with *P* < 0.05.

## Supplementary information

supplementary figure

Supplementary table

## Data Availability

All other data are available from the corresponding author upon reasonable request.
