# Supplementary material for: p32 promotes melanoma progression and metastasis by targeting EMT markers, Akt/PKB pathway, and tumor microenvironment
Source: Cell Death Dis. 2021 Oct 28;12(11):1012. doi: 10.1038/s41419-021-04311-5 (PMC8553772; doi:10.1038/s41419-021-04311-5)
Supplement: Supplementary file 1 — supplementary figure [file 41419_2021_4311_MOESM1_ESM.pptx]

## Slide 1
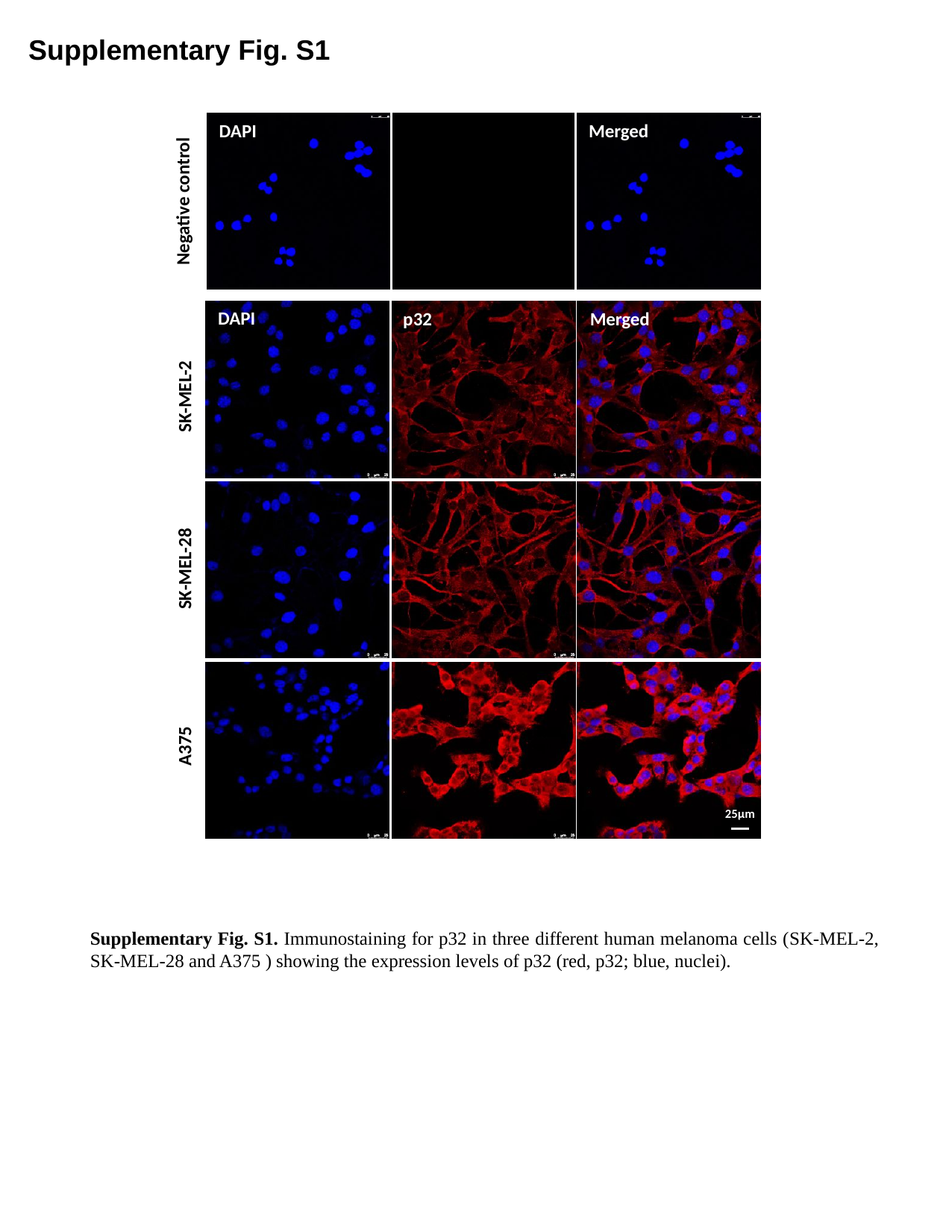

Supplementary Fig. S1
DAPI
Merged
Negative control
DAPI
p32
Merged
SK-MEL-2
SK-MEL-28
A375
25μm
Supplementary Fig. S1. Immunostaining for p32 in three different human melanoma cells (SK-MEL-2, SK-MEL-28 and A375 ) showing the expression levels of p32 (red, p32; blue, nuclei).

## Slide 2
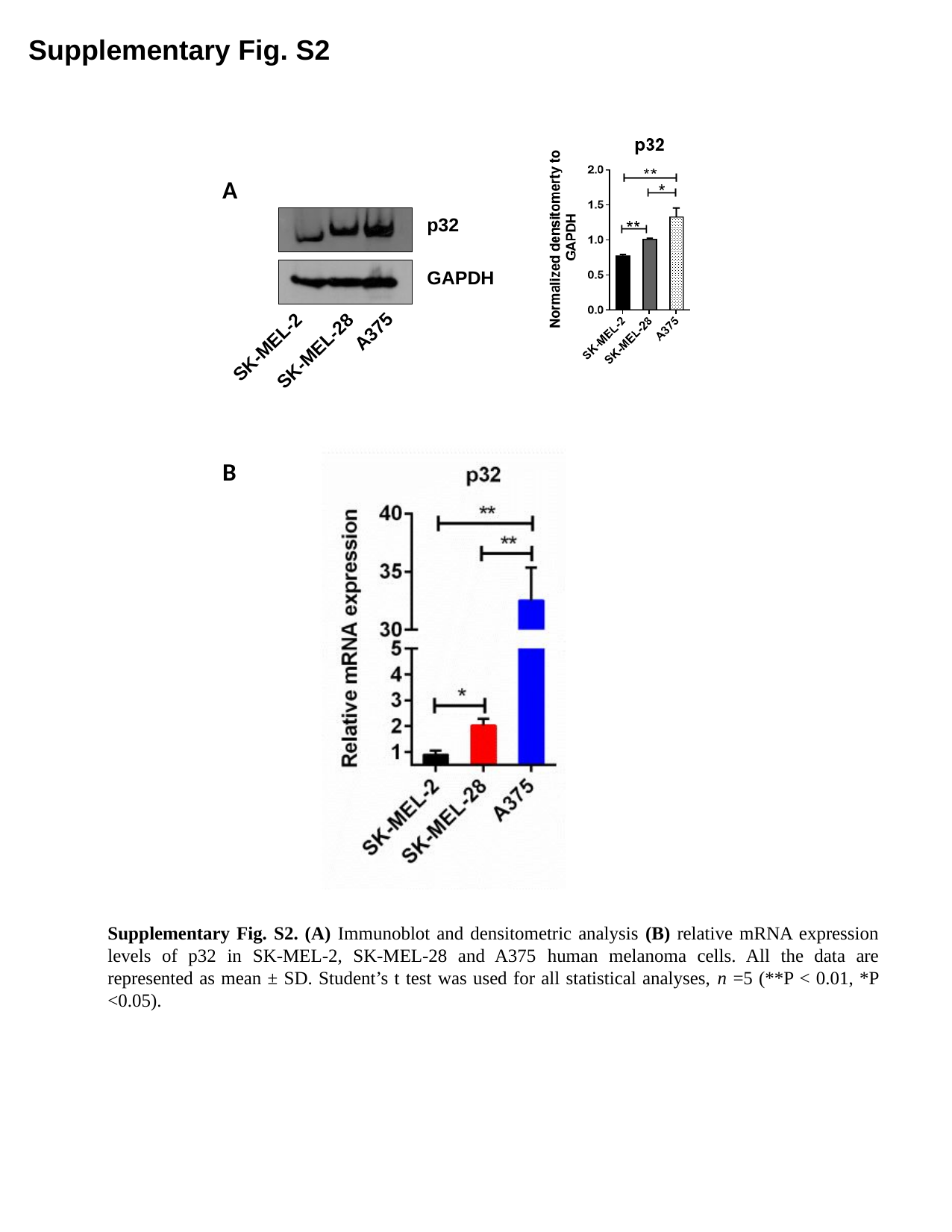

Supplementary Fig. S2
A
p32
GAPDH
A375
SK-MEL-2
SK-MEL-28
B
Supplementary Fig. S2. (A) Immunoblot and densitometric analysis (B) relative mRNA expression levels of p32 in SK-MEL-2, SK-MEL-28 and A375 human melanoma cells. All the data are represented as mean ± SD. Student’s t test was used for all statistical analyses, n =5 (**P < 0.01, *P <0.05).

## Slide 3
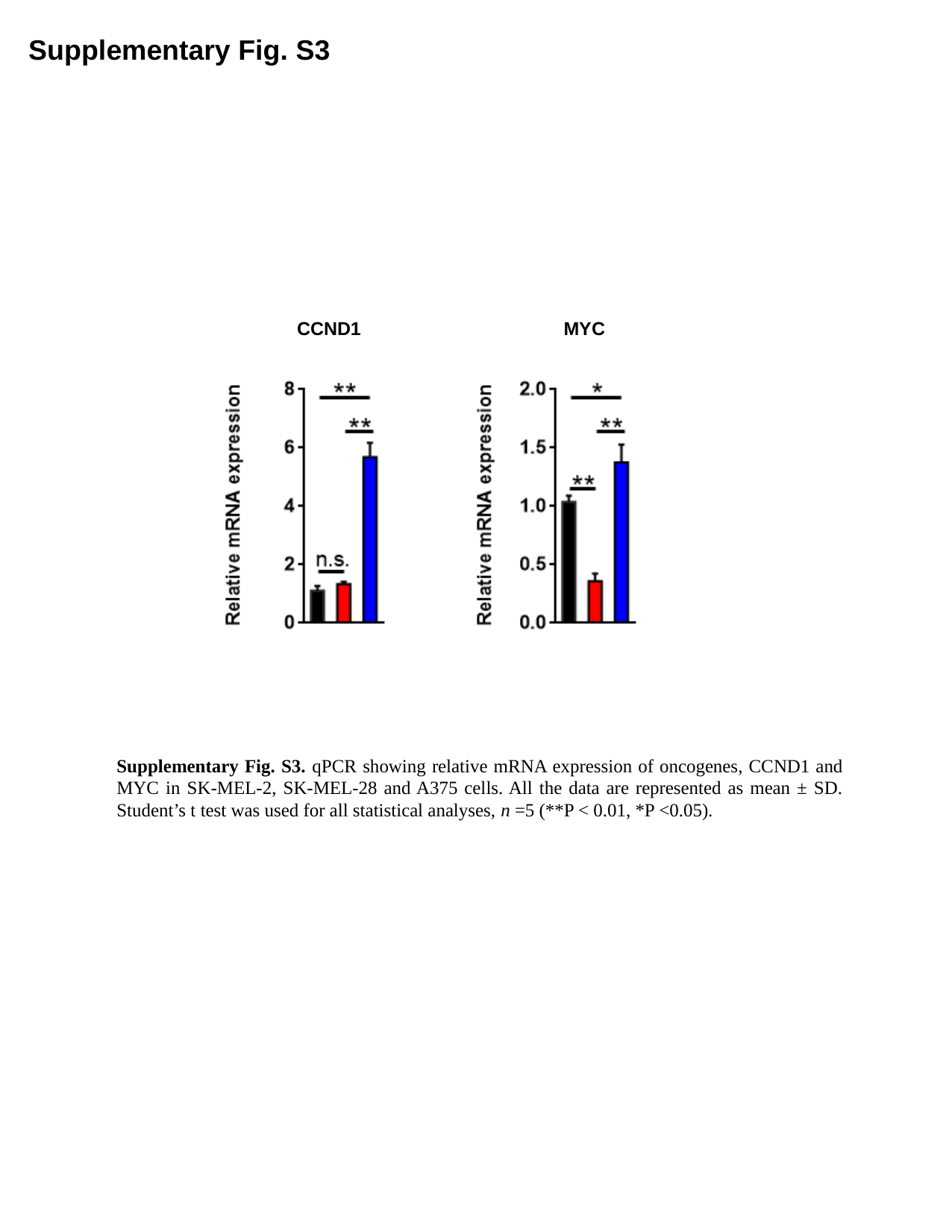

Supplementary Fig. S3
MYC
CCND1
Supplementary Fig. S3. qPCR showing relative mRNA expression of oncogenes, CCND1 and MYC in SK-MEL-2, SK-MEL-28 and A375 cells. All the data are represented as mean ± SD. Student’s t test was used for all statistical analyses, n =5 (**P < 0.01, *P <0.05).

## Slide 4
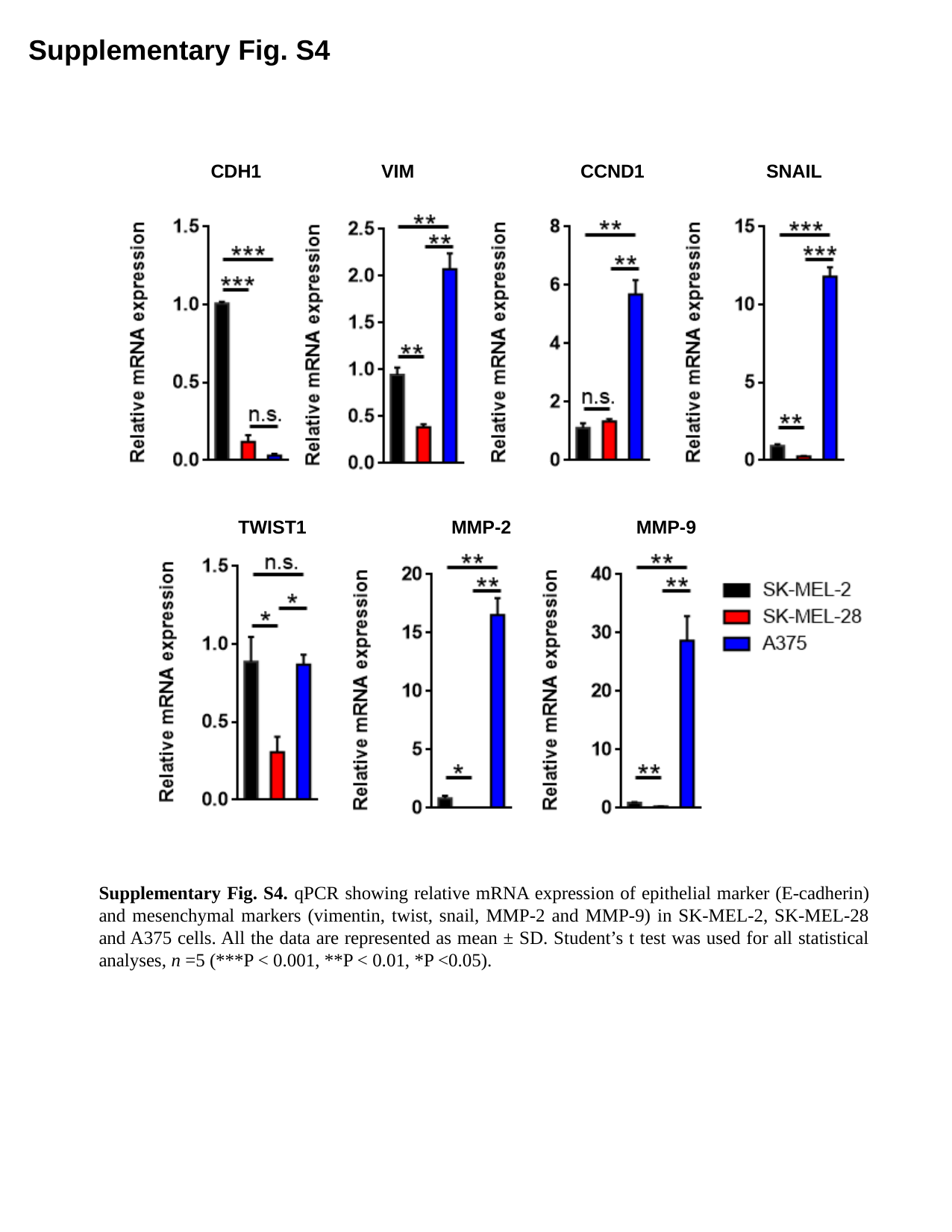

Supplementary Fig. S4
CDH1
VIM
CCND1
SNAIL
TWIST1
MMP-2
MMP-9
Supplementary Fig. S4. qPCR showing relative mRNA expression of epithelial marker (E-cadherin) and mesenchymal markers (vimentin, twist, snail, MMP-2 and MMP-9) in SK-MEL-2, SK-MEL-28 and A375 cells. All the data are represented as mean ± SD. Student’s t test was used for all statistical analyses, n =5 (***P < 0.001, **P < 0.01, *P <0.05).

## Slide 5
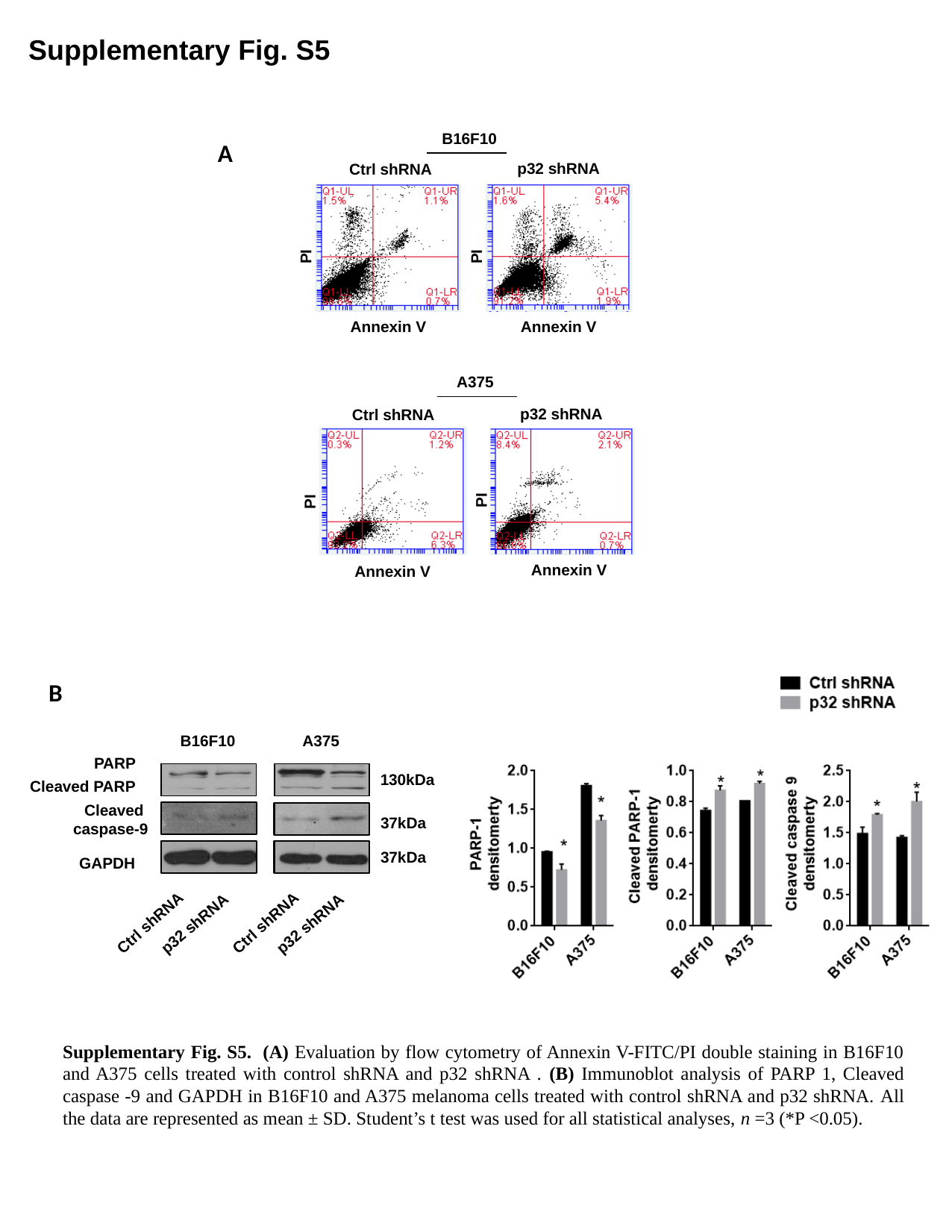

Supplementary Fig. S5
B16F10
p32 shRNA
Ctrl shRNA
Annexin V
PI
Annexin V
PI
A
A375
p32 shRNA
Ctrl shRNA
PI
Annexin V
PI
Annexin V
B
A375
B16F10
PARP
130kDa
Cleaved
caspase-9
37kDa
37kDa
GAPDH
Ctrl shRNA
Ctrl shRNA
p32 shRNA
p32 shRNA
Cleaved PARP
Supplementary Fig. S5. (A) Evaluation by flow cytometry of Annexin V-FITC/PI double staining in B16F10 and A375 cells treated with control shRNA and p32 shRNA . (B) Immunoblot analysis of PARP 1, Cleaved caspase -9 and GAPDH in B16F10 and A375 melanoma cells treated with control shRNA and p32 shRNA. All the data are represented as mean ± SD. Student’s t test was used for all statistical analyses, n =3 (*P <0.05).

## Slide 6
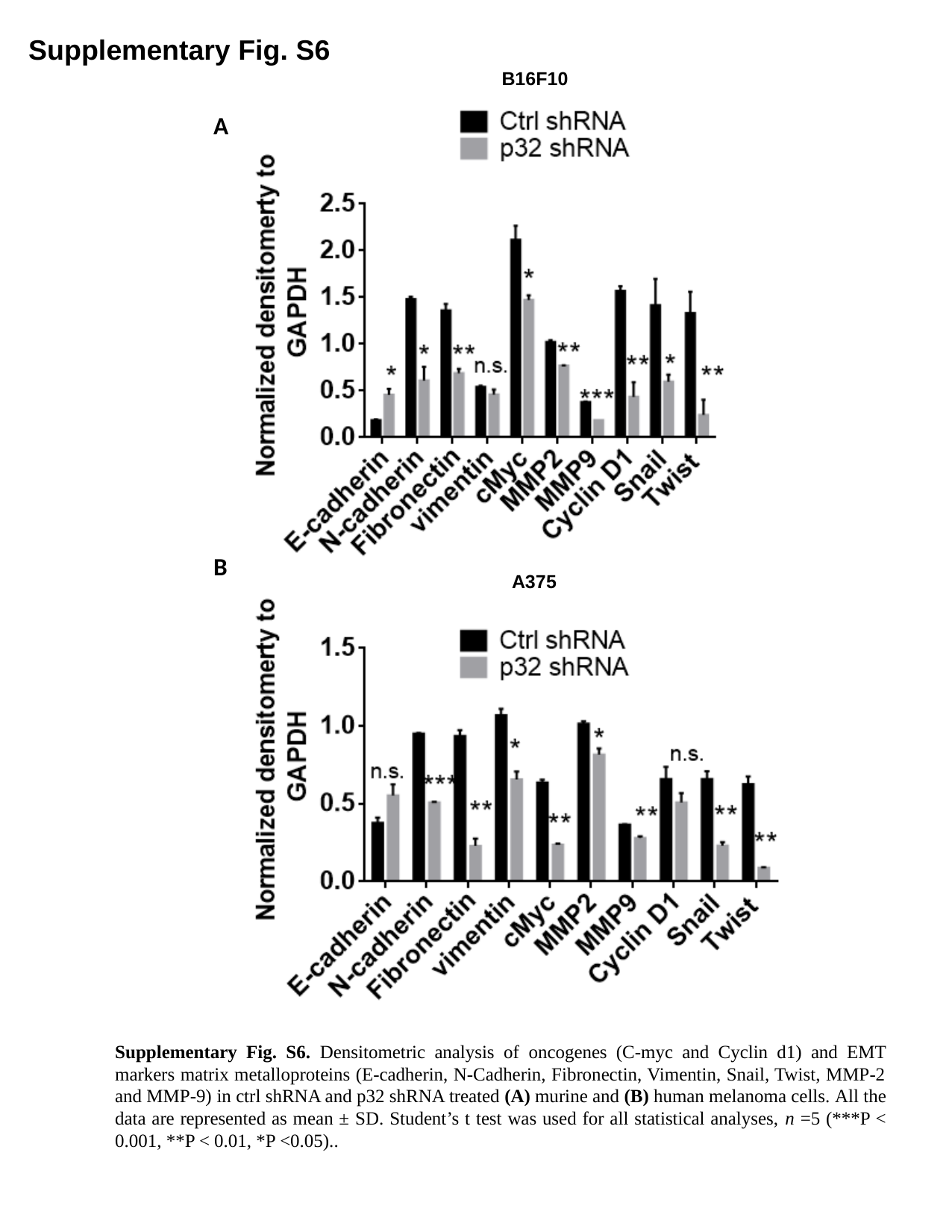

Supplementary Fig. S6
B16F10
A
B
A375
Supplementary Fig. S6. Densitometric analysis of oncogenes (C-myc and Cyclin d1) and EMT markers matrix metalloproteins (E-cadherin, N-Cadherin, Fibronectin, Vimentin, Snail, Twist, MMP-2 and MMP-9) in ctrl shRNA and p32 shRNA treated (A) murine and (B) human melanoma cells. All the data are represented as mean ± SD. Student’s t test was used for all statistical analyses, n =5 (***P < 0.001, **P < 0.01, *P <0.05)..

## Slide 7
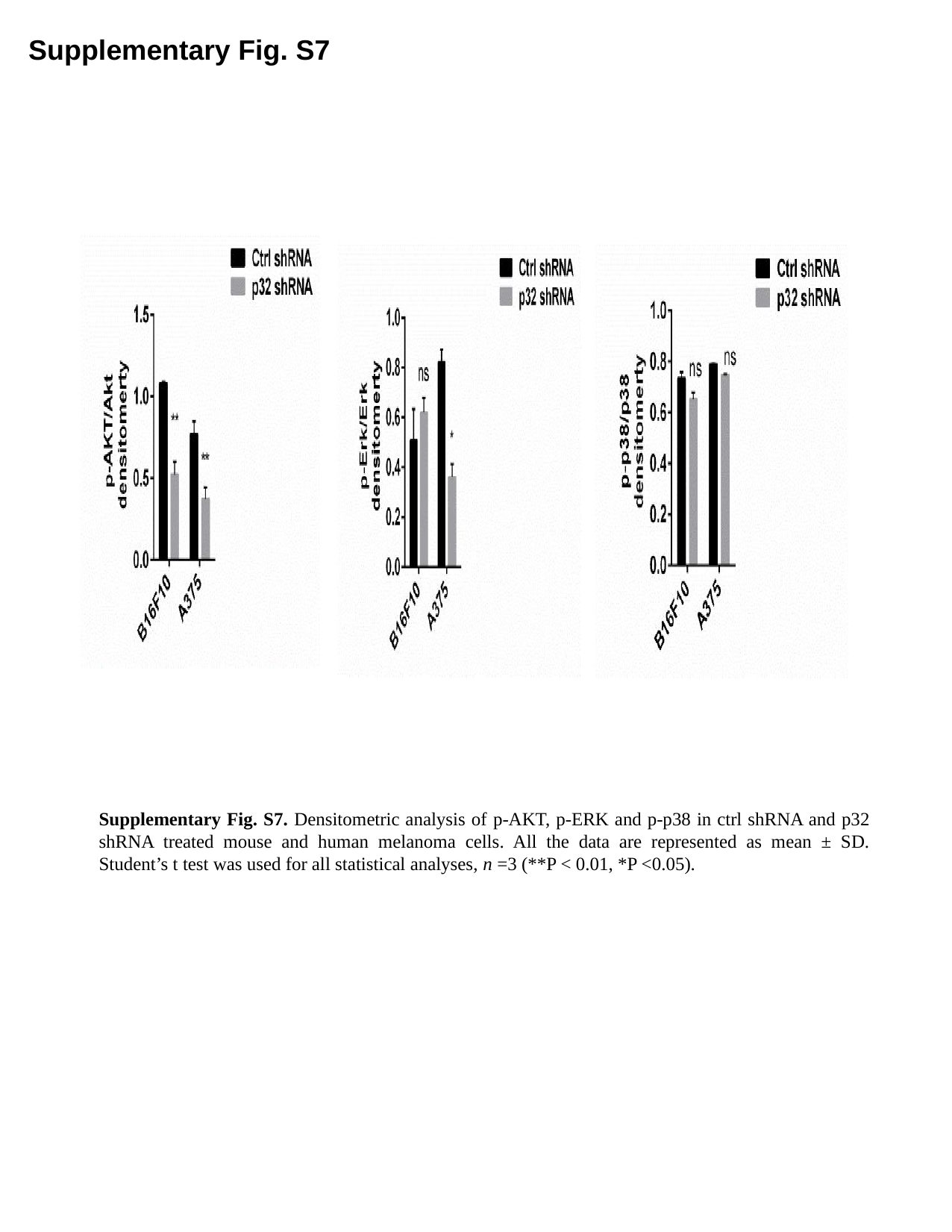

Supplementary Fig. S7
Supplementary Fig. S7. Densitometric analysis of p-AKT, p-ERK and p-p38 in ctrl shRNA and p32 shRNA treated mouse and human melanoma cells. All the data are represented as mean ± SD. Student’s t test was used for all statistical analyses, n =3 (**P < 0.01, *P <0.05).

## Slide 8
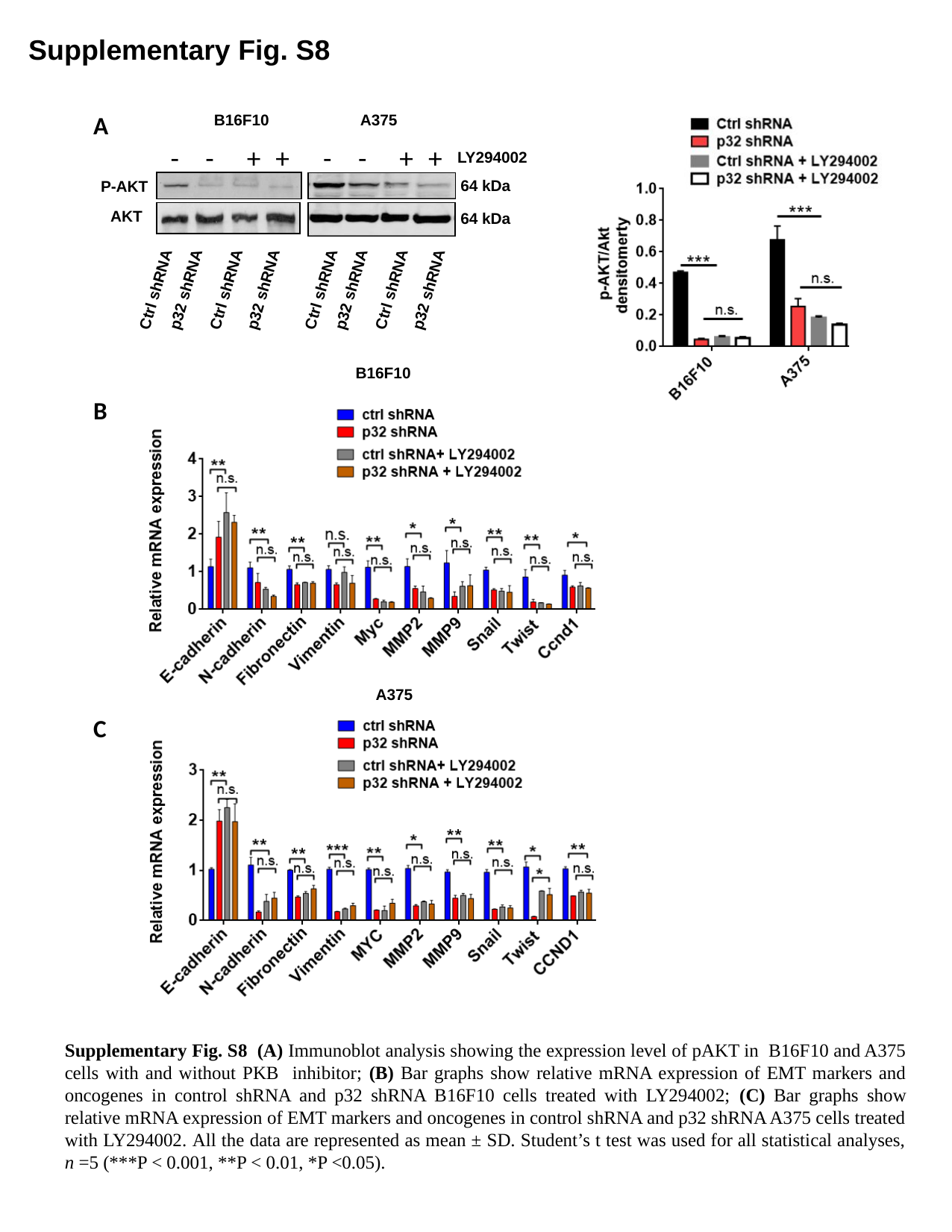

Supplementary Fig. S8
A
A375
B16F10
64 kDa
P-AKT
AKT
64 kDa
-
-
+
+
-
-
+
+
p32 shRNA
p32 shRNA
p32 shRNA
p32 shRNA
Ctrl shRNA
Ctrl shRNA
Ctrl shRNA
Ctrl shRNA
LY294002
B16F10
B
A375
C
Supplementary Fig. S8 (A) Immunoblot analysis showing the expression level of pAKT in B16F10 and A375 cells with and without PKB inhibitor; (B) Bar graphs show relative mRNA expression of EMT markers and oncogenes in control shRNA and p32 shRNA B16F10 cells treated with LY294002; (C) Bar graphs show relative mRNA expression of EMT markers and oncogenes in control shRNA and p32 shRNA A375 cells treated with LY294002. All the data are represented as mean ± SD. Student’s t test was used for all statistical analyses, n =5 (***P < 0.001, **P < 0.01, *P <0.05).

## Slide 9
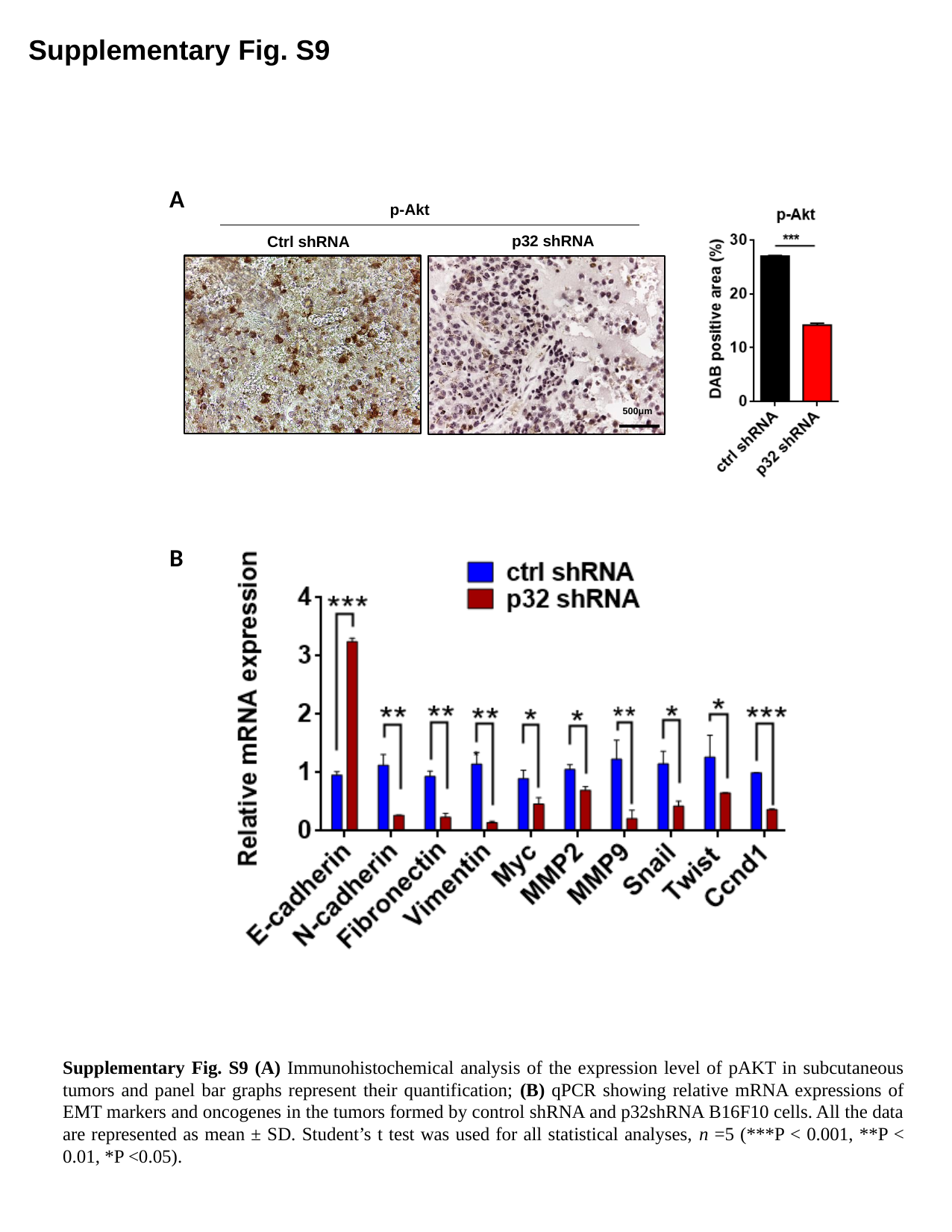

Supplementary Fig. S9
A
 p-Akt
 p32 shRNA
Ctrl shRNA
500μm
B
Supplementary Fig. S9 (A) Immunohistochemical analysis of the expression level of pAKT in subcutaneous tumors and panel bar graphs represent their quantification; (B) qPCR showing relative mRNA expressions of EMT markers and oncogenes in the tumors formed by control shRNA and p32shRNA B16F10 cells. All the data are represented as mean ± SD. Student’s t test was used for all statistical analyses, n =5 (***P < 0.001, **P < 0.01, *P <0.05).
