# Supplementary material for: p32 promotes melanoma progression and metastasis by targeting EMT markers, Akt/PKB pathway, and tumor microenvironment
Source: Cell Death Dis. 2021 Oct 28;12(11):1012. doi: 10.1038/s41419-021-04311-5 (PMC8553772; doi:10.1038/s41419-021-04311-5)
Supplement: Supplementary file 2 — Supplementary table [file 41419_2021_4311_MOESM2_ESM.docx]

**Supplementary Table 1.** Primers list for qPCR

| **Mouse** |  |  |
| --- | --- | --- |
| **Name** | **Forward primer** | **Reverse primer** |
| 18S FP | GTCTGTGATGCCCTTAGATG | AGCTTATGACCCGCACTTAC |
| MMP9 | AACATCTGGCACTCCACACC | GCAGAAGTTCTTTGGCCTGC |
| MMP2 | CTTCATCGCTGCACACCAGG | GGTATCTGGGCAGCAGAAAG |
| Vimentin | CTGAGGCTGCCAACCGGAACAA | CCTCGCCTTCCAGCAGCTTCC |
| snail | GGAAGCCCAACTATAGCGAGC | CAGTTGAAGATCTTCCGCGAC |
| Twist | CGGGTCATGGCTAACGTG | CAGCTTGCCATCTTGGAGTC |
| c1qbp | TTAGCTTTCAGGCCACTGGT | TCCGCAAGGAAATCCATTAG |
| N-Cadherin | TGAAACGGCGGGATAAAGAG | GGCTCCACAGTATCTGGTTG |
| E-cadherin | GGTTTTCTACAGCATCACCG | GCTTCCCCATTTGATGACAC |
| Fibronectin | CGAGGTGACAGAGACCACAA | CTGGAGTCAAGCCAGACACA |
| CyclinD1 | CTGGCCATGAACTACCTGGA | ATCCGCCTCTGGCATTTTGG |
| Myc | GCCCGCGCCCAGTGAGGATA | GCGGCGGCGGTGAGGTC |
| **Human** |  |  |
| C1QBP | AGTGCGGAAAGTTGCCGGGGA | GAGCTCCACCAGCTCATCTGC |
| 18S | GGCCCTGTAATTGGAATGAGT | CCAAGATCCAACTACGAGCTT |
| MMP9 | TTGACAGCGACAAGAAGTGG | GCCATTCACGTCGTCCTTAT |
| MMP2 | ATGACAGCTGCACCACTGAG | ATTTGTTGCCCAGGAAAGTG |
| Vimentin | AACAATGACGCCCTGCGCCA | TCTCATCCTGCAGGCGGCCA |
| snail | CCTAGCGAGTGGTTCTTC | CTGGAAGGTAAACTCTGGATT |
| Twist | CGGAGACCTAGATGTCATTG | ACGCCCTGTTTGTTTGAAT |
| E-cadherin | GTCACTGACACCAACGATAATCCT | TTTCAGTGTGGTGATTACGACGTTA |
| N-Cadherin | CACTGCTCAGGACCCAGAT | TAAGCCGAGTGATGGTCC |
| Fibronectin | CCGTGGGCAACTCTGTC | TGCGGCAGTTGTCACAG |
| MYC | TTCGGGTAGTGGAAAACCAG | CAGCAGCTCGAATTTCTTCC |
| CyclinD1 | GGATGCTGGAGGTCTGCGAGGAAC | GAGAGGAAGCGTGTGAGGCGGTAG |

**Supplementary Table 2.** List of Antibodies

| **Sl. No.** | **Antibody Name** | **Catalogue No.** | **Make** |
| --- | --- | --- | --- |
| 1. | Akt | 4685S | Cell Signalling |
| 2. | C -MYC Rabbit | 9402S | Cell Signalling |
| 3. | CD34 | SC-7324 | Santa-Cruz |
| 4. | CD45 | M0701 | Dako |
| 5. | Cyclin D1 | SC-753 | Santa Cruz |
| 6. | E –cadherin | SC-7870 | Santa Cruz |
| 7. | F 4/80 | SC-26643 | Santa-Cruz |
| 8. | GAPDH | 2118S | Cell Signalling |
| 9. | Ki67 | AB15580 | Abcam |
| 10. | p44/42 MAPK (ERK ½) | 9102S | Cell Signalling |
| 11. | Phospho Akt (Ser473)(D9E) | 4060S | Cell Signalling |
| 12. | Phospho-p44/42 MAPK (Er1/2) | 9106S | Cell Signalling |
| 13. | C1QBP Antibody (H-9) | sc-271200 | Santa Cruz |
| 14. | Snail (c15D3) | 3879S | Cell Signalling |
| 15. | Stat 3 (D3Z2G) | 12640S | Cell Signalling |
| 16. | Twist (twist2c1a) | SC-81417 | Santa Cruz |
| 17. | Vimentin(R28) | 3932S | Cell Signalling |
| 18. | Fibronectin | sc-8422 | Santa Cruz |
| 19. | N-cadherin Antibody | sc-8424 | Santa Cruz |
| 20. | PARP | 9542S | Cell Signalling |
| 21. | Caspase-9 | 9508S | Cell Signalling |
| 22. | MMP9 | sc-393859 | Santa Cruz |
| 23. | MMP2 | sc-13595 | Santa Cruz |
| 24. | Annexin V-FITC Apoptosis kit | 6592 | Cell Signalling |
